# Public Trust in Scientists for Cancer Information Across Political Ideologies in the US

**DOI:** 10.1001/jamanetworkopen.2025.46818

**Published:** 2025-12-04

**Authors:** Christopher W. Wheldon, Meghnaa Tallapragada, Erika L. Thompson

**Affiliations:** 1 Department of Social & Behavioral Sciences, College of Public Health, Temple University, Philadelphia, Pennsylvania; 2 Department of Advertising & Public Relations, Klein College of Media and Communication, Temple University, Philadelphia, Pennsylvania; 3 Department of Quantitative and Qualitative Health Sciences, University of Texas School of Public Health San Antonio, The University of Texas Health Science Center at San Antonio

## Abstract

**Importance:**

Public trust in scientists is essential for effective cancer communication and uptake of cancer control strategies. However, growing political polarization in the US may affect trust in scientific authorities.

**Objective:**

To determine whether political ideology is associated with trust in scientists as sources of cancer information, and whether individuals with more conservative political views would report lower levels of trust in scientists.

**Design, Setting, and Participants:**

This survey study is a cross-sectional secondary analysis of the 2024 Health Information National Trends Survey, a nationally representative survey conducted by the National Cancer Institute. This population-based sample of noninstitutionalized US adults was collected by mail using a stratified random sampling frame from March to September 2024. Respondents were aged 18 years or older and demographically diverse. The sample was weighted to be representative of the US adult population.

**Exposure:**

Political ideology measured on a 7-point bipolar scale from very liberal to very conservative.

**Main Outcome and Measure:**

Trust in scientists for cancer information, dichotomized as high (some or a lot) vs low (not at all or a little). Survey-weighted logistic regression models were used to estimate associations.

**Results:**

Of 7278 respondents (response rate, 27.3%), 6260 were included in the final analytic sample after excluding cases with missing data on the primary exposure or outcome. The mean age was 48.4 years (95% CI, 47.9-48.9 years). There were 52.1% men (95% CI, 51.2%-53.0%), and 35.1% reported a college degree (95% CI, 34.5%-35.7%). Most respondents (5320 individuals ) reported high trust in scientists as sources of cancer information (86.0%; 95% CI, 84.4%-87.5%). In adjusted logistic regression models, each 1-point increase toward greater conservatism was associated with a 25% decrease in the odds of reporting high trust (adjusted odds ratio, 0.75; 95% CI, 0.68-0.83). Adjusted estimated probabilities of high trust decreased across the political spectrum, from 93.7% those identifying as liberal (95% CI, 88.3%-96.7%) to 70.5% among those identifying as very conservative (95% CI, 63.9%-76.4%).

**Conclusions and Relevance:**

In this survey study of US adults, overall trust in scientists as sources of cancer information was high. However, a clear ideological gradient suggests the need for tailored messaging and trusted messengers to reach politically diverse audiences with evidence-based cancer information.

## Key Points

**Question:**

Is political ideology a factor associated with an individual’s trust in scientists as sources of cancer information?

**Findings:**

In this survey study of 6260 US adults, overall trust in scientists was high (86.0%), but significantly lower among individuals with more conservative political views. Estimated probabilities of high trust ranged from 93.7% among liberal respondents to 70.5% among very conservative respondents.

**Meaning:**

These findings suggest that public trust in scientists remains high overall, but the ideological gradient underscores the need for communication strategies that engage politically diverse audiences.

## Teaser

This survey study investigates whether political ideology is associated with trust in scientiests as asources of cancer information in the US.

## Introduction

Public trust in scientific authorities is a foundational element of effective public health communication, especially in areas like cancer prevention, screening, and treatment. Scientists are among the most trusted sources of information,^1^ yet recent data suggest that trust in scientists may be increasingly shaped by political ideology.^2^

In the US, political polarization has widened across numerous domains of public life. Surveys show that conservative-leaning individuals tend to express more skepticism toward vaccinations and other health interventions, as was evident during the COVID-19 pandemic.^3^ Moreover, the COVID-19 pandemic instigated angst and anger against scientists and public health officials.^4^ This ideological gap has important implications for cancer communication, particularly when scientific recommendations are perceived as politically charged or when trust in the messenger is as influential as the message itself. While skepticism is a critical component of the scientific process, public trust in the available science and government by the public is critical for adoption of cancer-related public health interventions.

The present study used nationally representative data from the Health Information National Trends Survey to estimate public trust in scientists as sources of cancer information and examine its association with political ideology. The following hypothesis was tested: Individuals with more conservative political ideologies will report significantly lower levels of trust in scientists as sources of cancer information compared with individuals with more liberal political ideologies. Findings from this study can inform future cancer information communication strategies that are tailored to different segments of the US population.

## Methods

This survey study used data from the National Cancer Institute, Health Information National Trends Survey collected in 2024. Details on the survey methodology can be found elsewhere.^5^ This study was determined to be exempt from human participants research by the Temple University institutional review board. We adhered to the Strengthening the Reporting of Observational Studies in Epidemiology (STROBE) reporting guideline for cross-sectional studies.

The primary dependent variable was trust in scientists for cancer information, and the primary independent variable was political viewpoint. Respondents were asked “In general, how much would you trust information about cancer from scientists?” They responded on a 4-point unipolar scale from not at all to a lot. Trust was operationalized as high (some or a lot responses) and low (not at all or a little responses) as was done in previous research.^6^ They were also asked 1 question about their political ideology: “Thinking about politics these days, how would you describe your own political viewpoint?” Responses were recorded on a 7-point bipolar scale from very liberal (1) to very conservative (7) with a moderate midpoint. Several control variables were considered, including sociodemographics (age, birth sex, marital status, and educational attainment), previous cancer diagnosis (self and biological relatives), and trust in cancer information from a doctor (ie, not at all or a little vs a lot or some). Race and ethnicity were self-identified. Ethnicity response options were no, not of Hispanic, Latino/a, or Spanish origin; yes, Mexican/Mexican American/Chicano/a; yes, Puerto Rican; yes, Cuban; and yes, another Hispanic, Latino/a, or Spanish origin. For analysis, ethnicity was coded as Hispanic (any yes) vs non-Hispanic. Race response options were American Indian or Alaska Native, Asian Indian, Black or African American, Chinese, Filipino, Guamanian or Chamorro, Japanese, Korean, Native Hawaiian, Other Asian, Other Pacific Islander, Samoan, Vietnamese, or White. Respondents could select more than 1 race. Owing to small cell sizes, race was recategorized into 7 mutually exclusive groups: American Indian or Alaska Native, East Asian (Chinese, Japanese, and Korean), Southeast Asian (Filipino and Vietnamese); Black or African American, Pacific Islander (Native Hawaiian, Guamanian or Chamorro, Samoan, and Other Pacific Islander), White, and multiracial or other (Asian Indian, Other Asian, and respondents selecting 2 or more races). In this sample, no respondents identified as Pacific Islander alone; multiracial selections that included a Pacific Islander subgroup were classified as multiracial or other. Race and ethnicity are included in this study because racial and ethnic minority populations experience disparities in cancer incidence and outcomes in the US.

### Statistical Analysis

Data were analyzed with SAS statsitical software version 9.4 (SAS Institute) using survey-weighting procedures to adjust point estimates and standard errors for household nonresponse and noncoverage bias. The final analytic dataset was created by removing missing cases on the trust and political ideology variables. Bivariable and multivariable logistic regression models were used to regress a binary trust outcome (ie, high trust vs low trust) on political ideology.

Control variables were added to the multivariable model if they exhibited a bivariable association with trust and political ideology (eg, age, marital status, education, race, cancer diagnosis in biological relative, and trust in doctors’ cancer information). Hot-deck imputation was used to replace missing responses for the following variables: age, birth sex, marital status, educational attainment, and Hispanic ethnicity. All results were weighted and adjusted to represent the noninstitutionalized adult US population.

We estimated survey-adjusted probabilities of high trust for each of the 7 political-ideology categories from the bivariable SURVEYLOGISTIC model in SAS. Between-group differences were tested with a design-based omnibus type 3 *F* test, and Šidák-adjusted pairwise comparisons of least-squares means were conducted on the probability (ilink) scale. All tests were 2-sided with α = .05.

Sensitivity analyses examined alternative codings of both the exposure and the outcome. First, we redefined the outcome as highest trust (a lot) vs all other categories and reestimated all survey-weighted models. Second, we varied political ideology using a quadratic term, a 3-level specification (left-of-center, moderate, and right-of-center), and a coding that treated skipped and/or refused responses as a separate category. We also probed potential bias from item nonresponse using skip-pattern indicators. Statistical significance was prespecified as 2-sided α = .05. For odds ratios (ORs), 95% CIs that do not include 1.00 were considered statistically significant.

## Results

Of 7278 respondents (27.3% response rate), 208 were excluded for missing data on trust and 810 were excluded for missing data on political ideology variables, leaving 6260 adults (mean age, 48.4 years; 95% CI, 47.9-48.9 years) in the final sample. There were 52.1% (95% CI, 51.2%-53.0%) men, and 35.1% (95% CI, 34.5%-35.7%) reported a college degree. Complete sample characteristics are reported in the Table. Most respondents rated high trust in cancer information from scientists (86.0%; 95% CI, 84.4%-87.5%); however, there was a significant inverse association between conservative political ideology and trust. In the bivariable model, higher scores on political ideology (ie, indicating greater political conservatism) were associated with a 27% decline in the odds of high trust in scientists for cancer information (OR, 0.73; 95% CI, 0.66-0.81). This estimate remained largely unchanged after adjusting for control variables, including age, educational attainment, family member with history of cancer, and trust in doctors for cancer information (adjusted OR,  0.75; 95% CI, 0.68-0.83). In the multivariable model, older age was associated with lower trust; college education and trust in cancer information from doctors were associated with higher trust.

**Table.** Analysis of Trust in Scientists as Sources of Cancer Information, Health Information National Trends Survey 7

Variable	Respondents, % (95% CI) (N = 6260)			OR (95% CI)	
	Total sample	Low trust	High trust	Bivariable	Multivariable adjusted^a^
Total	NA	14.0 (12.5-15.6)	86.0 (84.4-87.5)	NA	NA
Political ideology score, mean (95% CI)^b^	4.0 (4.0-4.1)	4.7 (4.5-4.9)	3.9 (3.8-4.0)	0.73 (0.66-0.81)	0.75 (0.68-0.83)
Age, mean (95% CI), y	48.4 (47.9-48.9)	52.2 (49.7-54.6)	47.8 (47.2-48.4)	0.99 (0.98-1.00)	0.98 (0.98-0.99)
Sex					
Female	47.9 (47.1-48.8)	14.1 (12.2-16.0)	85.9 (84.0-87.8)	1.00 [Reference]	NA
Male	52.1 (51.2-52.9)	14.0 (11.8-16.2)	86.0 (83.8-88.2)	1.01 (0.80-1.28)	NA
Marital status					
Not married	43.2 (42.2-44.6)	14.5 (11.5-17.4)	85.5 (82.6-88.5)	1.00 [Reference]	NA
Married or partnered	56.6 (55.4-57.8)	13.7 (11.8-15.6)	86.3 (84.4-88.2)	1.07 (0.78-1.45)	NA
Education					
High school or less	26.2 (24.7-27.7)	19.0 (15.6-22.3)	81.0 (77.7-84)	1.00 [Reference]	1.00 [Reference]
Some college	38.7 (37.1-40.2)	16.3 (13.1-19.5)	83.7 (80.5-86.9)	1.21 (0.87-1.68)	1.24 (0.88-1.76)
College graduate	20.7 (19.4-22.0)	9.5 (7.2-11.8)	90.5 (88.2-92.8)	2.23 (1.54-3.23)	1.86 (1.28-2.72)
Postgraduate	14.4 (13.3-15.5)	5.5 (3.8-7.2)	94.5 (92.8-96.2)	4.02 (2.74-5.90)	2.89 (1.86-4.48)
Hispanic ethnicity					
Hispanic	16.8 (16.3-17.4)	13.7 (9.8-17.6)	86.3 (82.4-90.2)	1.03 (0.73-1.47)	NA
Non-Hispanic	83.2 (82.6-83.7)	14.1 (12.5-15.7)	85.9 (84.3-87.5)	1.00 [Reference]	NA
Race^c^					
American Indian	1.1 (0.6-1.5)	6.9 (0.5-13.2)	93.1 (86.8-99.5)	2.06 (0.73-5.85)	NA
Black	11.5 (11.0-12.1)	18.2 (13.0-23.4)	81.8 (76.6-87.0)	0.69 (0.47-1.00)	NA
East Asian	2.2 (1.5-2.9)	11.8 (1.9-21.7)	88.2 (78.3-98.1)	1.14 (0.40-3.28)	NA
Southeast Asian	0.70 (0.34-1.06)	19.8 (0-45.8)	80.2 (54.22-100.0)	0.62 (0.08-5.05)	NA
White	73.0 (71.9-74.1)	13.2 (11.5-14.9)	86.8 (85.1-88.5)	1.00 [Reference]	NA
Multiracial or other	11.4 (10.1-12.7)	15.8 (11.5-20.1)	84.2 (79.9-88.5)	0.81 (0.58-1.14)	NA
Cancer history					
No personal history	90.4 (90.1-90.8)	13.4 (11.8-15.0)	86.6 (85.0-88.2)	1.00 [Reference]	NA
Personal history	9.6 (9.2-9.9)	19.9 (14.6-25.3)	80.1 (74.7-85.4)	0.62 (0.43-0.90)	NA
Family cancer history					
No family history	34.2 (32.6-35.8)	16.7 (13.3-20.0)	83.3 (80.0-86.7)	1.00 [Reference]	1.00 [Reference]
Family history	64.2 (62.4-65.9)	12.6 (11.0-14.2)	87.4 (85.8-89.0)	1.39 (1.04-1.87)	1.19 (0.85-1.66)
Missing	1.6 (1.1-2.2)	15.5 (6.8-24.1)	84.5 (75.9-93.2)	1.10 (0.53-2.27)	1.51 (0.62-3.68)
Trust in doctors for cancer information^d^					
Low trust	5.8 (4.6-6.9)	67.2 (59.2-75.1)	32.8 (24.9-40.8)	1.00 [Reference]	1.00 [Reference]
High trust	94.2 (93.1-95.4)	10.8 (9.6-11.9)	89.2 (88.1-90.4)	16.95 (11.86-24.21)	17.14 (10.88-27.00)

Abbreviations: OR, odds ratio; NA, not applicable.

^a^ Multivariable models used listwise deletion for 24 cases missing trust in doctors, yielding 6236 respondents. The model was adjusted for age, educational attainment, and cancer diagnosis in biological relative, which all exhibited bivariable associations with trust and political ideology.

^b^ Political ideology responses were recorded on a 7-point bipolar scale from very liberal (1) to very conservative (7) with a moderate midpoint.

^c^ Race categories are mutually exclusive. East Asian includes Chinese, Japanese, and Korean. Southeast Asian includes Filipino and Vietnamese. Multiracial and other includes Asian Indian, Other Asian, and respondents selecting 2 or more races; in this sample, no respondents identified as Pacific Islander. Hispanic ethnicity was coded as Hispanic vs non-Hispanic; original Health Information National Trends Survey options were Cuban; Mexican and/or Mexican American and/or Chicano/a; Puerto Rican; and another Hispanic, Latino/a, or Spanish origin.

^d^ There were 24 missing cases. The denominator was 6236.

Adjusted estimated probabilities of high trust in scientists for cancer information decreased across the political spectrum (Figure), from 93.7% (95% CI, 88.3%-96.7%) among liberal to 70.5% (95% CI, 63.9%-76.4%) among very conservative respondents. Between group differences were statistically significant (*F*_6,49_ = 11.37; *P* < .001). Šidák-adjusted pairwise contrasts showed higher survey-adjusted probabilities for very liberal vs very conservative, liberal vs very conservative, somewhat liberal vs conservative, somewhat liberal vs very conservative, moderate vs conservative, and and moderate vs very conservative, and somewhat conservative vs very conservative. In sensitivity analyses, results were directionally consistent with the alternative coding for the primary outcome (eg, some/a lot vs not at all/a little), for political ideology, and when considering nonresponse bias. Across these analyses, effect size directions and inferences were unchanged.

**Figure.**
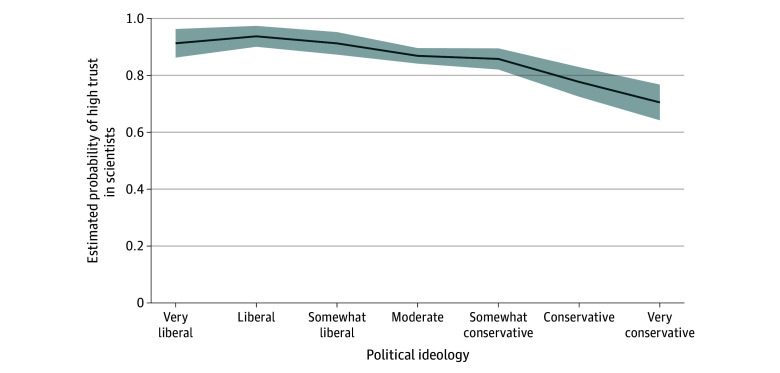
Estimated Probability of High Trust in Cancer Information From Scientists Across Political Ideologies Solid lines denote mean probabilities, and shaded areas denote 95% CIs.

## Discussion

This survey study found that trust in scientists as sources of cancer information was generally high among US adults, with 86.0% reporting some or a lot of trust. While political ideology was a factor significantly associated with trust—showing a clear gradient from higher trust among liberal individuals to lower trust among conservative individuals—trust levels remained relatively strong across the ideological spectrum. Even among those identifying as very conservative, over 70% expressed high trust in scientists for cancer information.

Independent of political ideology, lower trust among older adults may reflect cohort differences in media use and prior institutional experiences that encourage caution, whereas higher trust with college education may reflect greater familiarity with scientific norms and confidence in evaluating evidence. The association with trust in doctors points to source-credibility effects and trust transfer from clinician messengers to science-facing sources.^7^ Clinician-scientists comessaging, using plain-language, nondigital formats with transparent explanations of uncertainty may help extend clinician credibility to scientific content.

Our findings underscore important implications for science communication. First, scientists have an important role in public health communication regarding cancer risk, prevention, screening recommendations, and treatment advances. Second, there is a need to identify trusted messengers who can connect with ideologically diverse audiences on these issues in a manner that communicates the key cancer messaging for prevention or treatment. Third, this work serves as a call to action for the scientific community to prioritize science literacy in public health communication.

Notably, National Cancer Institute–designated Cancer Centers have a mandate to reduce the burden of cancer through outreach, education, and equitable access to evidence-based prevention and care strategies in their catchment areas.^8^ Our findings suggest that effective communication requires more than information dissemination. The contact hypothesis suggests that trust can be strengthened, especially when viewed as an outgroup, when scientists avoid hierarchical dynamics, emphasize cooperation, and foster supportive dialogue.^9^ Moreover, when people are confronted with their mortality, as is often the case with cancer communication, the Terror Management Health Model suggests that they may in the short term respond by trying to manage the immediate threat by either avoiding it or by taking action to address it, while over time their cultural worldviews become more influential.^10^ Thus, effective cancer communication should also involve compassion-based, respectful communication that acknowledges people’s fears and affirms their values. Strengthening public trust through such strategic communication can enhance the reach and impact of cancer communication efforts.

### Limitations

There are important limitations from this study to consider. The overall response rate was low, which increases potential for nonresponse bias, although analytic weights were applied to minimize this impact. In addition, trust and political ideology were assessed using single-item measures, which may not capture the full complexity or multidimensionality of these constructs. Additional research is needed to examine trends in these associations over time.

## Conclusions

In this survey study of US adults, overall trust in scientists as sources of cancer information was high, despite being collected during a time (ie, 2024) of heightened political polarization. The data showed that scientists remained broadly trusted figures in cancer communication. This resilience in public trust provides a valuable foundation for continued efforts to promote science-based information and reduce the burden of cancer across all communities.

## Notes

Supplement. Data Sharing Statement
