# Supplementary material for: Public Trust in Scientists for Cancer Information Across Political Ideologies in the US
Source: JAMA Netw Open. 2025 Dec 4;8(12):e2546818. doi: 10.1001/jamanetworkopen.2025.46818 (PMC12679320; doi:10.1001/jamanetworkopen.2025.46818)
Supplement: Supplement. — Data Sharing Statement [file jamanetwopen-e2546818-s001.pdf]

Data Sharing Statement

Wheldon. High Public Trust in Scientists for Cancer Information Across Political Ideologies. *JAMA Netw Open*. Published December 04, 2025. doi:10.1001/jamanetworkopen.2025.46818

Data
Data available: No

Additional Information
Explanation for why data not available: Data is publicly available at https://hints.cancer.gov/
